# Co-Solvent Exfoliation of Hexagonal Boron Nitride: Effect of Raw Bulk Boron Nitride Size and Co-Solvent Composition

**DOI:** 10.3390/nano10061035

**Published:** 2020-05-28

**Authors:** Xiang Nie, Guo Li, Zhao Jiang, Wei Li, Ting Ouyang, Jianfeng Wang

**Affiliations:** 1 College of Materials Science and Engineering, Hunan University, Changsha 410082, China; kan.nx@foxmail.com (X.N.); liguomaster@163.com (G.L.); jiangzhao@hnu.edu.cn (Z.J.); liwei5168@hnu.edu.cn (W.L.); wangjianfeng@hnu.edu.cn (J.W.); 2 Hunan Province Key Laboratory for Advanced Carbon Materials and Applied Technology, Hunan University, Changsha 410082, China

**Keywords:** liquid phase exfoliation, boron nitride nanosheets, co-solvent cluster, size effect

## Abstract

Exfoliation of two-dimensional boron nitride nanosheets (BNNSs) from parent bulk material has been receiving intensive attention because of its fascinating physical properties. Liquid exfoliation is a simple, scalable approach to produce single-layer or few-layer BNNS. In this paper, water/propanol co-solvent exfoliation of bulk boron nitride under the assistance of sonication was investigated in detail. Special attention was paid on the effect of raw bulk boron nitride size and co-solvent composition. The results show that sonication of small-size hexagonal boron nitride tends to generate large nanosheets, due to a predominant solvent wedge effect. In addition, it is found that the composition of water/propanol co-solvent has an important effect on exfoliation efficiency. Interestingly, although two isomers of 1-propanol (NPA) and 2-propanol (IPA) have the same molecular weight and similar surface tension, their aqueous solutions allow the formation of boron nitride nanosheets dispersion with markedly different concentrations. It is proposed that due to their spatial configuration difference, NPA with its longer molecular chain and fewer hydrophobic methyl group tends to form dynamic water-NPA clusters with larger size than water-IPA clusters. The hydrodynamic radius of the co-solvent “clusters” was calculated to be 0.72 nm for water/NPA system and 0.44 nm for water/IPA system at their maximum, respectively. Their size changes, represented by two curves, indicate a strong “cluster size” effect on exfoliation efficiency. Our work provides an insight into co-solvent exfoliation of hexagonal boron nitride and emphasizes the importance of co-solvent cluster size in exfoliation efficiency.

## 1. Introduction

Since the first successful isolation of monolayered graphene in 2004, two-dimensional nanomaterials have witnessed an explosive growth in research interest [1,2] due to their exceptional properties for many promising applications. As a structural analogue of graphene, hexagonal boron nitride nanosheets (BNNSs) consist of sp^2^-bonded atomic layers where B and N atoms are alternatively arranged into a hexagonal lattice. The regular atomic arrangement permits high phonon velocity and low phonon scattering, leading to an excellent thermal conductive property (~2000 W∙m^−1^∙K^−1^) [3]. ENREF 1 Unlike the highly symmetrical C–C bonds in graphene, B and N atoms have different electronegativity, which causes the localization of π electrons and confers excellent electrical-insulating properties to BNNSs (wide band gap of 5–7 eV, low dielectric constant of 3–4) [4,5]. These unique properties make BNNSs a very competitive candidate for thermal management in electronic devices [6,7].

Liquid exfoliation is considered to be a scalable route to derive BNNSs from the parent bulk hexagonal boron nitride (*h*-BN). It is proved that liquid exfoliation facilitates the applications of BNNSs in substrate deposition [8], films [9,10], composites [11,12], nanodevices [13] and detectors [14]. Among various liquid exfoliation techniques, ultrasonic-assisted exfoliation is frequently used because of its versatility, low cost and the access it provides to high quality BNNSs [15,16]. Essentially, ultrasonic-assisted exfoliation is a process in which the van der Waals interactions between the atomic layers of bulk boron nitride are overcome by ultrasonic energy, and the detached individual nanosheets are subsequently stabilized by solvent molecules. With the ultrasonic vibration [17] as a driving force, the strong affinity of solvent molecules on the surface of *h*-BN is necessary. In this case, a minimization of their interfacial energy [16,18,19] is essential to achieve efficient exfoliation. Up to now, a large number of organic solvents have been explored for liquid exfoliation process of *h*-BN, including nonvolatile solvents, such as N-methyl-2-pyrrolidone, dimethyl-formanmide, dimethyl sulfoxide [20,21,22], and volatile solvents such as chloroform, isopropyl alcohol, *t*-butyl alcohol, etc. [23,24,25]. As the most abundant solvent resource on Earth, water is also found to assist with exfoliation and dispersion of BNNSs [26,27]. Among the solvents, IPA is preferred for liquid exfoliation of *h*-BN due to its high exfoliation efficiency, low toxicity, and easy removal during subsequent applications [16]. Besides the pursuit of a single solvent system, recently, attention has been paid to mixtures of solvents for improving the exfoliation and dispersion of *h*-BN [18,25]. The mixed solvents strategy combines the advantages of individual solvents and it can even turn two “poor” solvents into “good” solvents, such as ethanol and water [28]. Among the various mixtures of solvents, co-solvents consisting with water and organic solvents are considered a promising route for exfoliation of *h*-BN because of their reduced cost, improved safety and easy scalability [28,29], However, as the number of solvents and possible solvent mixtures is numerous, it is important to establish a methodology to choose appropriate solvents/co-solvents and probe the related liquid exfoliation mechanisms.

Some important parameters of solvents, such as surface tension, Hildebrand solubility parameters, Hansen solubility parameters and molecular weight were proposed to choose appropriate solvents/co-solvents [19,29,30,31]. It was considered that a lower surface tension difference between *h*-BN and solvent would facilitate the exfoliation and dispersion. It is determined experimentally that an optimal surface tension for an individual solvent is 35 mJ∙m^−2^, generating the most effective exfoliation and dispersion for *h*-BN [19]. However, for co-solvents with easily tuneable surface tension (by varying the components ratio), it is found out that the optimal surface tension value is variable, dependent on the type of selected co-solvents. For example, the optimal surface tensions of co-solvents of water with *tert*-butanol, isopropanol, 1-propyl alcohol, ethanol, methanol, and monoethanolamine are 21.3, 24.5, 25.3, 28, 32.9, and 55 mJ∙m^−2^ respectively [29,32]. In this case, surface tension is not the sole factor that influences the effectiveness of co-solvent exfoliation. Shen et al. [33,34] proposed that the matching of the ratio of two components of surface tension, i.e., polar and dispersive, can be used to determine exfoliation efficiency of co-solvents. Unfortunately, the authors have not treated the mismatched value for *h*-BN in detail. Marsh et al. [29] and Halim et al. [16] suggested that solvent molecular size might play a more important role than surface tension. However, most of their works were performed on comparing solvents with different molecular weight. For isomers with same molecular weight, their differences in exfoliation efficiency are barely researched. The same issue arises when using Hildebrand solubility parameter and Hansen parameters [18], which does not take account of solvent sizes. Despite those efforts, the details of underlying mechanisms for co-solvent exfoliation of *h*-BN still lack understanding and require more systematic studies.

In this work, *h*-BN is exfoliated in a frequently used water/propanol co-solvent mixture with the assistance of ultrasonication. Aiming to explore the fundamental factors governing the process and probe into the underlying mechanism, we investigated the effect of process variables such as centrifuge speed, sonication time, raw *h*-BN size, and co-solvent composition. In particular, the influence of co-solvent composition on exfoliation efficiency is discussed in detail based on the comparison of two co-solvents using propanol isomers, which are water/1-propanol (NPA) and water/2-propanol (IPA). Despite a similar surface tension and the same molecular weight, the two co-solvent systems present different exfoliation behaviors. Their differences in molecular structure and solvent-solvent interaction were inspected in detail. A new concept of co-solvent cluster size as an important solvent parameter is proposed. Altering the ratio of water to propanol in co-solvent causes the change of cluster size and thus influence exfoliation efficiency. We believed that co-solvent cluster size effect provides an insight into co-solvent exfoliation of hexagonal boron nitride, and it could extend our understanding of co-solvent exfoliation processes for other two dimensional materials.

## 2. Materials and Methods

### 2.1. Materials

All chemical reagents were of analytical grade and were obtained commercially. *h*-BN powder (2 µm) was purchased from Hongwu Materials Technology Co., Ltd. (Guangzhou, China). *h*-BN powder (30 µm) was purchased from Tianyuan Junrong Chemical New Materials Co., Ltd. (Liaoning, China). 1-propanol (NPA) and 2-propanol (IPA) were purchased from Aladdin Reagent Co., Ltd. (Shanghai, China).

### 2.2. Methods

In a typical process concerning the effect of component ratio on liquid exfoliation efficiency, the co-solvent solution was prepared by mixing IPA or NPA with water in a volume fraction of 0%, 20%, 40%, 50%, 60%, 80%, 100%, respectively. Two g of weighed *h*-BN powder were added to 400 mL of the co-solvents and sonicated for 3 h using an JP-040S sonicator (Skymen, Shenzhen, China) at a power of 240 W and a frequency of 40 kHz. After standing overnight, the suspension was centrifuged at 3000 rpm for 22 min (LG10-2.4A centrifuge, LingLi, Beijing, China), and decanted immediately to collect the supernatants. To account for the concentration of BNNSs, 100 mL of supernatants was taken and vacuum filtered using a pre-dried microporous filter membrane (0.22 µm). The weight of pre-dried microporous filter membrane is *m*_1_. The filtered BNNSs on the pre-weighted filter was dried and weighted to be *m*_2_. Thus the concentration *c* of BNNS in every 100 mL supernatants was determined to be:

(1) $$c=\frac{m_{2}-m_{1}}{100}$$

Processing parameters including ultrasonic time and centrifugation speed were varied to learn the effects on concentration and size distribution of BNNSs using co-solvent of 50 vol.% 2-propanol.

### 2.3. Characterizations

The morphology and microstructure of raw materials and exfoliated nanosheets were observed using field emission scanning electron microscopy (SEM, S4800, Hitachi, Tokyo, Japan) and transmission electron microscopy (TEM, G260-300, Titan, Hillsboro, OR, USA). Raman spectra was acquired at a spectral range of 1500–2500 cm^−1^ using an inVa Raman spectrometer (Renishaw inVia, London, UK) equipped with a laser wavelength of 532nm. X-ray diffraction (XRD, MiniFlex, Tokyo, Japan) patterns were collected in the scan range of 10°–90° with scanning speed of 5°/min. The functional group was analysed using Fourier transformation infrared spectroscopy (FTIR, iS10, Nicolet, Madison, WI, USA. The average particle size and size distribution of BNNSs in dispersion was characterized using a dynamic light scattering nanoparticle size analyser (DLS, Zetasizer Nano ZS, Malvern Panalytical, Malvern, UK). The specific surface area was obtained using Brunauer-Emmett-Teller (BET) equation, and nitrogen adsorption-desorption isotherms were performed using an ASAP 2460 system (Micrometrics, Norcross, GA, USA) at 77 K.

### 2.4. Statistical Analysis

Statistical analysis was performed using a one-way ANOVA method by SPSS 17.0 software (IBM, Armonk, NY, USA. Probability level of *p* < 0.01 was considered statistically significant. All data were expressed as mean ± standard deviation (SD).

## 3. Results and Discussion

### 3.1. Effect of Raw h-BN Size

It is recognized that the size of BNNSs has a strong influence on the mechanical, and thermal properties of their macroscopic materials [10,35]. So far, most of studies on size control focused on manipulating the processing parameters such as exfoliation time, centrifuge speed and time [10]. Few works have taken into account the influence of raw bulk materials. However, the size of employed bulk *h*-BN varied from one to several tens of microns [8,36], which often lead to the differences in crystallinity, stacking order and defects. These differences on inter-layer interaction would generate an effect on exfoliation efficiency. In order to clarify the size effect of *h*-BN on the resultant BNNSs, *h*-BN raw materials with a size of 2 µm and 30 µm are chosen for comparison.

**Figure 1:**
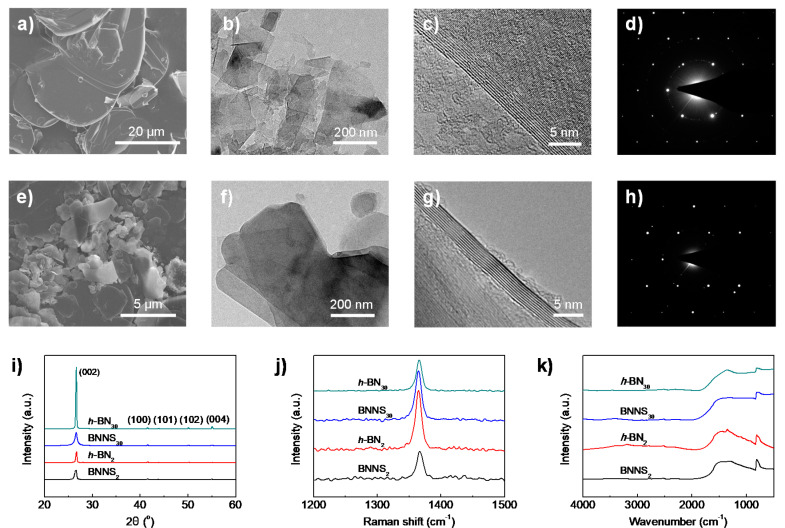
Morphology and microstructure of raw hexagonal boron nitride (*h*-BN) materials and exfoliated boron nitride nanosheets (BNNSs). Scanning electron microscopy (SEM) images of (**a**) *h*-BN_30_ and (**e**) *h*-BN_2_ raw materials. Transmission electron microscopy (TEM) images of (**b**–**d**) BNNS_30_ and (**f**–**h**) BNNS_2_. The diffraction spots in TEM (**d**,**h**) showed a typical hexagonal lattice. (**i**) X-ray diffraction (XRD), (**j**) Raman spectroscopy, and (**k**) Fourier transformation infrared spectroscopy (FTIR) spectroscopy of pristine *h*-BN and exfoliated BNNSs.

The large size difference of raw *h*-BN_30_ and *h*-BN_2_ materials (subscripts 30 and 2 denote the average size of *h*-BN in microns) is clearly revealed from SEM observation (Figure 1a,e). The lateral size of *h*-BN_30_ is found in the range of 20–40 µm, while *h*-BN_2_ only has a size from several hundred nanometres to a few microns. Exactly the same liquid exfoliation procedures (water/IPA co-solvent = 50/50 vol.%, ultrasonic time: 48 h, centrifugation speed: 3000 rpm) were applied to the two raw materials. Interestingly, from the TEM observations (Figure 1b,f), it is found that BNNSs obtained from *h*-BN_30_ (referred to BNNS_30_) have much smaller lateral sizes than those exfoliated from *h*-BN_2_ (referred to BNNS_2_), which are 100–250 nm and 200–500 nm, respectively. The results indicate that a larger *h*-BN does not necessarily produce larger-sized BNNSs. The thicknesses of the two BNNSs, on the other hand, show no obvious difference, which are typically around 8–9 layers (Figure 1c,g). Their selected area electron diffractions (SAED) (Figure 1d,h) exhibit typical six-fold symmetry patterns, indicating that the ultrasonic process does not destroy the in-plane lattice of BNNSs.

The structural difference of the two raw *h*-BNs and the derived BNNSs were characterized by XRD, Raman spectra and FTIR. A sharp (002) peak with considerably high intensity at 2θ = 26.66° is presented in XRD curve for *h*-BN_30_ (Figure 1i). The intensity is much higher and obvious narrower than that for *h*-BN_2_, indicating that *h*-BN_30_ has a higher crystal integrity and crystallinity. After exfoliation, both BNNSs present reduced peak intensity and increased peak width. Moreover, the peak position slightly shifts to lower angle comparing to their parent *h*-BNs, indicating the presence of few-layer BNNSs. The interlayer distance calculated using Bragg’s law increases from 0.3341 nm for *h*-BN_30_ to 0.3343 nm for BNNS_30_. Similarly, the interlayer distance increases from 0.3336 nm for *h*-BN_2_ to 0.3343 nm for BNNS_2_. The slightly increased inter-layer distance of BNNSs suggests that the exfoliated BNNSs have a less extended/ordered stacking in the *c*-axis direction.

A typical Raman signature is found for bulk *h*-BNs and exfoliated BNNSs as a prominent peak in the region of 1364–1366 cm^−1^ (Figure 1j), which is assigned to the E_2g_ vibration mode of the *h*-BN. It is seen that the peak intensity of BNNS_2_ is notably decreased comparing to its raw materials *h*-BN_2_, indicating a reduction of layer numbers [37] and weaker interaction between layers [38]. However, the Raman spectra showed an opposite trend for *h*-BN_30_ and BNNS_30_, which suggests that the latter is thicker than the former. This might be due to the excessive agglomeration of BNNS_30_ after drying. FTIR spectra (Figure 1k) shows the characteristic absorption peak at around 1366 cm^−1^, which can be attributed to the out-of-plane bending vibration of B–N–B. The characteristic absorption peak around 813 cm^−1^ is attributed to the in-plane stretching vibration of B–N. No obvious peak shift is observed because ultrasonic exfoliation is a simple physical process without functionalization of BNNS.

**Figure 2:**
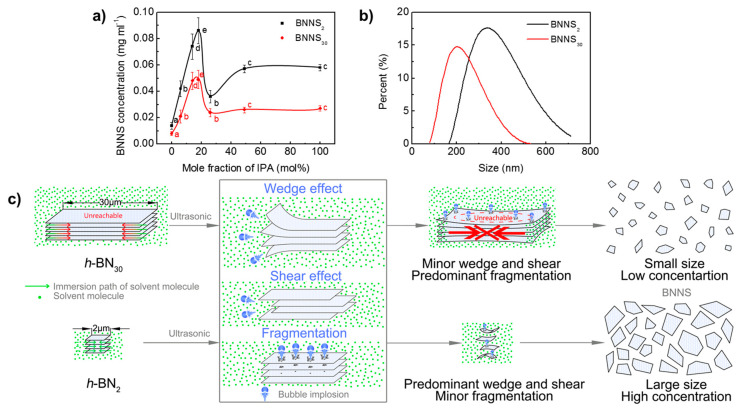
Effect of raw *h*-BN size on exfoliation. (**a**) Change of BNNS concentration with co-solvent formulations. (**b**) Size distribution of BNNSs. (production conditions: exfoliation time of 48 h, centrifugal speed of 3000 rpm, and 19 mol% IPA). (**c**) Schematic diagram of the effect of raw *h*-BN size on liquid exfoliation efficiency. Data presented as means ± SD. A = 0.01, from small to large: a → e.

The size of *h*-BN has an obvious effect on the resultant BNNS concentration and sizes. *h*-BN_30_ and *h*-BN_2_ are exfoliated in the water/IPA co-solvent with different IPA mole fractions by ultrasonic irradiation (time: 3 h, centrifugation speed: 3000 rpm), and the dependence of the resultant BNNS concentration on IPA mole fraction is shown in Figure 2a. It is seen that the concentrations of BNNS_30_ and BNNS_2_ have a similar evolution trend. With the increase of IPA mole fraction, their concentrations increase first, reach maximum values at around 19 mol% IPA, and then decrease. With IPA mole fraction further increases from 49 mol% to pure IPA, the exfoliated concentration shows no significant difference. Differently, the concentration of BNNS_2_ is higher than that of BNNS_30_, suggesting that *h*-BN_30_ is more difficult to exfoliate than *h*-BN_2_. The maximum concentration of BNNS_2_ is 0.086 mg/mL, which is nearly two times of that of BNNS_30_ (0.049 mg/mL). In addition, the hydrodynamic diameter of BNNS_30_ and BNNS_2_ with the same exfoliation conditions (ultrasonic time: 48 h, centrifugal speed: 3000 rpm, IPA fraction: 19 mol%) is measured by DLS and compared, as shown in Figure 2b. It is seen that BNNS_30_ has a much smaller value and narrower span (79–531 nm), compared with BNNS_2_ (164–712 nm), consistent with the observation by TEM.

Specific surface areas were experimentally determined to indirectly confirm the thickness difference of layered materials giving the same weight [39]. While *h*-BN_2_ and *h*-BN_30_ has only 7.75 and 1.33 m^2^/g, after exfoliation, apparent increase in specific surface area were found for BNNS_2_ and BNNS_30_ as 39.12 and 77.46 m^2^/g, respectively (Figure S1a,b). This supports the successful exfoliation of *h*-BN as thinner layers provide higher specific surface area. The comparison with BNNS_2_ and BNNS_30_ provides a strong indication that BNNS_30_ are smaller and/or thinner than BNNS_2_. The smaller size of BNNS_30_ is confirmed from DLS results. The thickness of their stacks, however, does not show much difference from TEM observation. Their similarity in stack thickness can also been implied from the pore size distribution (PSD) obtained from nitrogen adsorption isotherms by applying nonlocal density functional theory (NLDFT) carbon slit pore model [40,41]. They showed almost identical pore space periodicity (Figure S1c) only with different pore volume. According to Guo et al.’s model [41] depicting slot pores created in irregular stacking of multilayer graphene, X = 3.28 nm is determined from pore space periodicity, as the stack thickness, for both BNNSs. The corresponded 9–10 layers is in consistent with the observation in TEM. The higher cumulative pore volume (Figure S1d) of BNNS_30_ than BNNS_2_, on the other hand, indicates its higher specific surface area.

Based on the abovementioned results, the effect of raw *h*-BN size on exfoliation is proposed as shown in Figure 2c. During ultrasonic irradiation, “fragmentation”, “shear” and “wedge” effects are generated, due to the implosion of micrometre-sized bubbles [42,43]. For *h*-BN_30_ with large lateral size and high crystallinity, it is hard to achieve sufficient immersion and insertion of solvent molecules, leading to weak “wedge” and “shear” interactions. Therefore, the “fragmentation” effect is predominant and thus small nanosheet dispersion with low concentration are produced. For *h*-BN_2_ with small size, co-solvent molecules can relatively easily insert into the stacks and weaken the van der Waals interactions between layers [44]. In this case, “wedge” and “shear” effects are predominant, leading to large nanosheet dispersion with high concentration.

### 3.2. Effect of Centrifugation Speed and Ultrasonic Time

**Figure 3:**
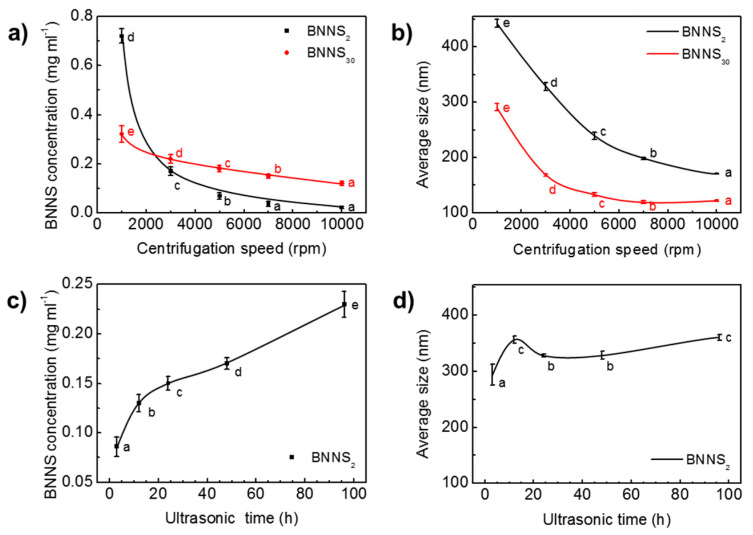
Effect of centrifugation speed on (**a**) BNNS concentration and (**b**) average size. Effect of ultrasonic time on (**c**) BNNS concentration and (**d**) average size. Data presented as means ± SD. α=0.01, from small to large: a → e.

The extraction of BNNSs from *h*-BN by ultrasound contains two successive steps of ultrasonic treatment and centrifugation. Processing parameters, such as ultrasonic time, and centrifugation speed have strong influence on the resultant BNNS concentration and size [42,45]. Figure 3 presents the variation of BNNS concentration and average size with centrifugation speed and ultrasonic time under constant IPA fraction of 19 mol%. As the speed increased from 1000 rpm to 3000 rpm, the concentration of BNNS_2_ quickly dropped from 0.72 mg/mL to 0.17 mg/mL (Figure 3a). The nearly 76% drop indicates that a great number of multilayered, large-sized BNNSs were screened out during the high-speed centrifugation process. Similarly, the concentration of BNNS_30_ decreased from 0.32 mg/mL at 1000 rpm to 0.22 mg/mL at 3000 rpm. As the rotation speed surpasses 3000 rpm, both BNNS_2_ and BNNS_30_ concentrations begin to level off with a slow decrease. BNNS_30_ has a higher concentration than BNNS_2_ at high centrifugation speed range from 3000 rpm to 10,000 rpm. It shows that from 7000 rpm to 10,000 rpm, effect of centrifugation speed on BNNS_2_ concentration is of little significance, while for BNNS_30_ the effect is still of significance. It is due to the predominant fragmentation effect during sonicating *h*-BN_30_ that generates a large amount of small-sized nanosheets with high dispersion stability (Figure 2b). Higher centrifugation speeds are required to achieve BNNS_30_ size separation, while for BNNS_2_ with larger sizes, a relatively low centrifugation speed is sufficient to screen out a majority of the nanosheets, leaving less to be separated under higher centrifugation speed.

Along with the drop of BNNS concentration, the size of BNNSs decreases with the increased centrifugation speed (Figure 3b), as high speed centrifugation works as an effective separation of BNNS with different sizes in suspension [45,46]. For BNNS_2_, the average size decreases from 442 nm at 1000 rpm to 171 nm at 10,000 rpm. A decrease from 291 nm at 1000 rpm to 122 nm at 10,000 rpm is observed for BNNS_30_. The average size of BNNS_2_ is always higher than that of BNNS_30_, consistent with the TEM observation.

The influence of ultrasonic time on the concentration and size of BNNSs is shown in Figure 3c,d, respectively. It is seen that concentration first increases sharply within 12 h, and then increases in a relatively slow rate. The average size of BNNS_2_, on the other hand, shows an increase from 294 nm to 357 nm as ultrasonic irradiation time increases from 3 h to 12 h. When the ultrasound exposure time was increased from 12 h to 96 h, the average size of BNNS_2_ fluctuated slightly between 328 nm and 361 nm. This indicates that, after 12 h, ultrasonic irradiation time has little influence on the average size of BNNSs. Similarly, in Du et al.’s work, it was found that the thickness of BNNS decreased with the increase of ultrasonic time, but after a certain period of ultrasonic irradiation time, the BNNS thickness no longer decreases and it fluctuates within a certain range [47]. It is most likely that the liquid exfoliation process is a dynamic process in which the large BNNSs are continuously exfoliated off, and the large BNNSs are continuously fragmented into smaller and thinner pieces, due to the presence of ultrasonic waves [48].

### 3.3. Effect of Co-Solvent Compositions

**Figure 4:**
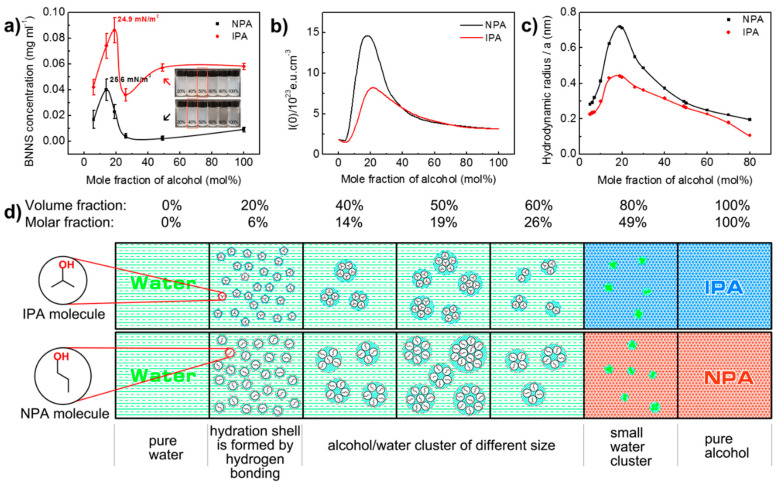
Analysis of water/1,2-propanol co-solvent exfoliation of *h*-BN into BNNSs. (**a**) The influence of water/1,2-propanol co-solvent formulations on concentration of exfoliated BNNSs. Digital photos in this figure are exfoliated BNNS suspensions in 10 mL glass bottles (volume fraction is marked on bottles). (**b**) Dependence of the zero-angle X-ray scattering intensity on different formulations of water/1,2-proponal co-solvent. Adapted with permission from [53]. Copyright (1990) American Chemical Society. (**c**) Hydrodynamic radius of water/1,2-propanol co-solvent clusters. (**d**) Schematic diagram of the evolution of water/1,2-propanol co-solvent clusters.

To further probe into the effect of alcohol/water co-solvents ratio on liquid exfoliation efficiency, water/NPA system was introduced as a reference. *h*-BN_2_ was used as the raw material. Ultrasonic irradiation time and centrifugation speed are set to be 3 h and 3000 rpm, respectively. The change of BNNS concentration with the water/alcohol ratio is shown in Figure 4a. In both systems, the BNNS concentration first increases with the addition of alcohol. After a maximum value is reached, the concentration begins to fall until it reaches a plateau at high mole fraction of alcohol. It is obvious that water/IPA has better exfoliation efficiency than water/NPA by producing higher BNNS concentration at each alcohol/water ratio. For water/IPA co-solvent, the optimal BNNS exfoliation occurs at 19 mol% IPA, generating a 0.086 mg/mL BNNS dispersion. However, for water/NPA system, the highest BNNS concentration is only 0.040 mg/mL at 14 mol% of NPA. The corresponding surface tension is 24.9 mJ m^−2^ for the water/IPA and 25.6 mJ m^−2^ for water/NPA, which is in close agreement with the reported value by Marsh et al. (24.5 mJ m^−2^ for IPA and 25.3 mJ m^−2^ for NPA) [29]. It should be noted that BNNS concentration obtained using water/IPA in our study is more than two times of that using water/NPA system, while in Marsh et al.’s work, not much difference was shown in their results on UV-vis absorption intensity for the two systems. This is probably due to the fact that we directly measured the weight of BNNSs in suspensions for comparison and in order to obtain measurable BNNS weight, a larger volume of solution (100 mL) is required comparing to a cuvette volume of only 3–4 mL for UV-vis test, so any difference presented by the two systems can be accumulated and uncovered.

It was proposed previously that the relative molecular weight associated with solvent molecular size and surface tension have an effect on the exfoliation efficiency of *h*-BN in co-solvents. However, NPA and IPA are typical isomers with the same molecular weight, yet their differences lie not only on exfoliated BNNS concentration but also the optimal alcohol/water ratio. As such, we pay attention to the influence of solvents’ molecular structure and their molecular interactions with water.

NPA and IPA are typical mono-hydroxylated alcohols miscible with water in any concentration range. Macroscopically, their aqueous solutions do not present any phase separation. However, due to the formation and breakage of hydrogen bonding between water and alcohol, they dynamically form agglomerates in nanoscale, presenting a microheterogeneity [49,50,51]. The size and concentration of the formed agglomerates (clusters) in the alcohol/water mixture are varied with the size and shape of the alcohol molecular chains and their compositions. Therefore, they have been recognized as the reason for anomalous behaviour compared with their pure component [52] and been characterized using various techniques [53,54,55,56]. For instance, due to the steric hindrance effect of molecular clusters on electromagnetic waves, the propagation of electromagnetic waves is disturbed, so co-solvent system with different compositions exhibits different elastic light scattering intensity and x-ray diffraction intensity. The higher the intensity, the stronger the microheterogeneity. Figure 4b shows adapted results of the zero-angle X-ray scattering intensity of water/1,2-propanol co-solvent at different alcohol contents [53]. It is very interesting to find that zero-angle X-ray curves of two systems displayed a similar profile with BNNS concentration curves presented in Figure 4a. The zero-angle X-ray intensity of alcohol aqueous solutions peaked at about 15 mol% NPA and about 20 mol% IPA, which are very close to the alcohol concentration of exfoliation peak (14 mol% for NPA and 19 mol% for IPA). Such agreement implies that the formation and properties of alcohol/water cluster have imposed an influence on exfoliation effect. Similar pattern can also been found on elastic light scattering and solution viscosity results of water/1,2-propanol co-solvent with different alcohol contents [52,57].

As zero-angle X-ray intensity is a total reflectance both of size and concentration of clusters, it is hard to distinguish their effects on exfoliation behaviour of BNNS. In order to account for the size effect of water/alcohol cluster, the equivalent hydrodynamic radius was calculated using Stokes-Einstein relation as following [53,58]:

(2) $$D=\frac{k_{B}\cdot T}{6\pi \cdot \eta \cdot a}$$

where D is diffusion coefficient, *k_B_* is Boltzmann constant (1.38 × 10^23^ J/K), *T* is temperature of the fluid (298K), *η* is dynamic viscosity of the fluid, and *a* is hydrodynamic radius of spherical “Stokes” particle (in this article is co-solvent cluster). The Stokes-Einstein relation is used to describe the diffusion coefficient of a spherical “Stokes” particle undergoing Brownian motion in a fluid at uniform temperature [58]. Here, as the local structure of water and sufficiently hydrophobic groups (from alcohol molecules) is clathrate hydrates like [59,60], water/alcohol cluster can be dynamically considered as spherical “Stokes” particle. Therefore, the calculated hydrodynamic radius is used to define the “size” of cluster [61]. Diffusion coefficient [62] and viscosity [57] data for this calculation were taken from literature (Table S1). The calculated results were drawn in Figure 4c. These data show that the hydrodynamic radius of alcohol/water cluster quickly increased with the increase of the mole fraction of alcohol before the maximum value at 19 mol%, and then gradually decreased with the further increase of alcohol contents. The overall trends of the curves are very similar to the zero-angle X-ray intensity results. The maximum value of hydrodynamic radius is 0.72 nm for water/NPA system and 0.44 nm for water/IPA system at 19 mol%, respectively. The result is close to Großmann’s calculation for NPA solution [52] (0.8 nm at NPA % = 20 mol%). In all the alcohol mole fraction, the hydrodynamic radius of water/NPA cluster is always larger than that of water/IPA cluster. The results can be rationalized by the fact that NPA has longer molecular chain and fewer hydrophobic methyl group than IPA molecules. Longer molecular chain increases the size of clusters in space, and the number of hydrophobic groups will determine the intermolecular attraction within the cluster and thus affect the size of the cluster [63]. However, we should emphasize that the lifetime of water/1,2-propanol hydrogen bond is between 1 and 10 picoseconds, which means the existence of cluster is dynamic rather than structural in origin [59,64].

The resembles of Figure 4b,c and Figure 4a suggest that at least the size of alcohol/water cluster plays a critical role in BNNS exfoliation. As such, the formation and evolution process of water/1,2-propanol co-solvent clusters are proposed and illustrated in Figure 4d. When the NPA and IPA concentration in water increases to 6 mol%, a hydration shell is gradually formed around the alcohol molecules with the establishment of a hydrogen bond network under the envelope of water molecules [53,63]. Since the NPA chain is longer than the IPA molecule, the radius of the hydrated shell of NPA molecule is larger than that of IPA. With the increase of alcohol concentration, the hydration shell of NPA molecules is more likely to collide with each other, and agglomerate to form bigger co-solvent clusters. Thus, compared to the water/IPA system, a water/NPA solution presents much higher zero-angle X-ray diffraction intensity (Figure 4b), and the BNNS yield peak appears even earlier (Figure 4a). The size of both cluster peaks at 19 mol% alcohol, which is exactly the optimal composition to produce BNNSs for water/IPA. This seems reasonable as large-sized molecules are considered favourable for good BNNS dispersion [16,29]. However, the optimal co-solvent ratio for water/NPA is 14 mol% of NPA (Figure 4a), at which the cluster radius is smaller than its peak value. Combining the fact of larger-sized water/NPA cluster producing much less BNNSs, it is certified that only appropriate cluster size favours *h*-BN exfoliation and BNNS stabilization, giving an optimal BNNS concentration. When alcohol concentration continues to increase to about 50 mol%, it is difficult to form hydration shell with alcohol molecules due to too few water molecules, therefore only small water clusters form in the solution, and the exfoliation effect is close to that of pure solvent.

The solvent size effect on the stability of exfoliation has been evaluated for several layered materials including *h*-BN [49,65]. It was suggested that solvents with larger molecular size facilitate the exfoliation by preventing interlayer Leonard-Jones attraction. However, the molecular size comparison mainly performed on solvents with different mole mass [16,29,66]. For isomers with same mole mass such as NPA and IPA, we proved that the formation of water-alcohol clusters plays an important role, and it exhibits “size” effect. Clusters with too large size are difficult to penetrate the interlayer gap between *h*-BN, and clusters of too small size cannot play a good steric hindrance role in the interlayer gap. Only the clusters with appropriate size can hinder overlap and agglomeration of BNNSs after ultrasonic microbubble explosion breaks the interlayer van der Waals force of *h*-BN.

## 4. Conclusions

In summary, the liquid exfoliation process of *h*-BN using water/1,2-propanol systems present a notable “size” effect in terms of raw material and co-solvent clusters. As microstructural characterizations confirmed, larger sized *h*-BN possessed much higher crystal integrity, making it difficult to exfoliate during liquid exfoliation process. Different exfoliation effects were thus applied, and smaller BNNSs were produced. Co-solvents using water and propanol isomers produced a much higher exfoliation concentration than pure solvents alone. Although they possess the same molecular mass, IPA and NPA aqueous solutions exhibited different exfoliation efficiency. The BNNS yield using water/IPA solution is more than twice that obtained using NPA aqueous solution, and they peaked at different alcohol compositions, which are 19 mol% of IPA and 14 mol% of NPA. With little difference in their surface tension, the formation of water-alcohol clusters was proposed to rationalize the results. Due to their different spatial configuration, NPA with its longer molecular chain and fewer hydrophobic methyl group tends to form dynamic water-NPA clusters with larger size than water-IPA clusters. The changes of “cluster size” with alcohol composition were simulated and it exhibited a good consistency with the BNNS concentration curves. This provided solid evidence on the “cluster size” effect of liquid exfoliation using alcohol aqueous solutions. Therefore, although solid-liquid interactions are essential to select solvents in the first place for liquid exfoliation, by using co-solvent systems, the solvent-solvent interaction is the key in further screening of high performance systems. To be specific, in this work the dynamic formation of water-alcohol clusters was a critical factor that substantially influences the output of liquid exfoliation of *h*-BN. Our work provides a rational strategy to select co-solvent systems by taking full consideration of the differences between isomers. It has subsequent scientific applications for the enlargement of the data library for liquid exfoliation of 2D layered materials.

## Supplementary Materials

The following are available online at https://www.mdpi.com/2079-4991/10/6/1035/s1, Figure S1: Nitrogen adsorption-desorption isotherm and specific surface areas of *h*-BN_2_, *h*-BN_30_, BNNS_2_ and BNNS_30_, Figure S2: Enlarged SEM images of *h*-BN_2_ and *h*-BN_30_, Table S1: The data and results of cluster size calculation.
