# Supplementary material for: Co-Solvent Exfoliation of Hexagonal Boron Nitride: Effect of Raw Bulk Boron Nitride Size and Co-Solvent Composition
Source: Nanomaterials (Basel). 2020 May 28;10(6):1035. doi: 10.3390/nano10061035 (PMC7352847; doi:10.3390/nano10061035)
Supplement: Supplementary file 1 [file nanomaterials-10-01035-s001.pdf]

nanomaterials

MDPI

support information

Co-solvent Exfoliation of Hexagonal Boron Nitride: Effect of Raw Bulk Boron Nitride Size and Co-Solvent Composition

Xiang Nie [1,2], Guo Li [1,2], Zhao Jiang [1], Wei Li [1,2], Ting Ouyang [1,2,*] and Jianfeng Wang [1]

1 College of Materials Science and Engineering, Hunan University, Changsha 410082, China; kan.nx@foxmail.com (X.N.); liguomaster@163.com (G.L.); jiangzhao@hnu.edu.cn (Z.J.); liwei5168@hnu.edu.cn (W.L.); wangjianfeng@hnu.edu.cn (J.W.)

2 Hunan Province Key Laboratory for Advanced Carbon Materials and Applied Technology, Hunan University, Changsha 410082, China

* Correspondence: oyt@hnu.edu.cn; Tel.: +86-159-7313-2768

Received: 25 April 2020; Accepted: 23 May 2020; Published: date

Experimental Section

Characterization

The specific surface area was obtained using Brunauer-Emmett-Teller (BET) equation, and nitrogen adsorption-desorption isotherms were performed using a Micrometrics ASAP 2460 at 77 K. The true density of $BNNS_2$ and $BNNS_{30}$ was measured by Accupyc 1340 helium pycnometer. The equilibrium rate is set to 0.005 psig/min.

Results Section

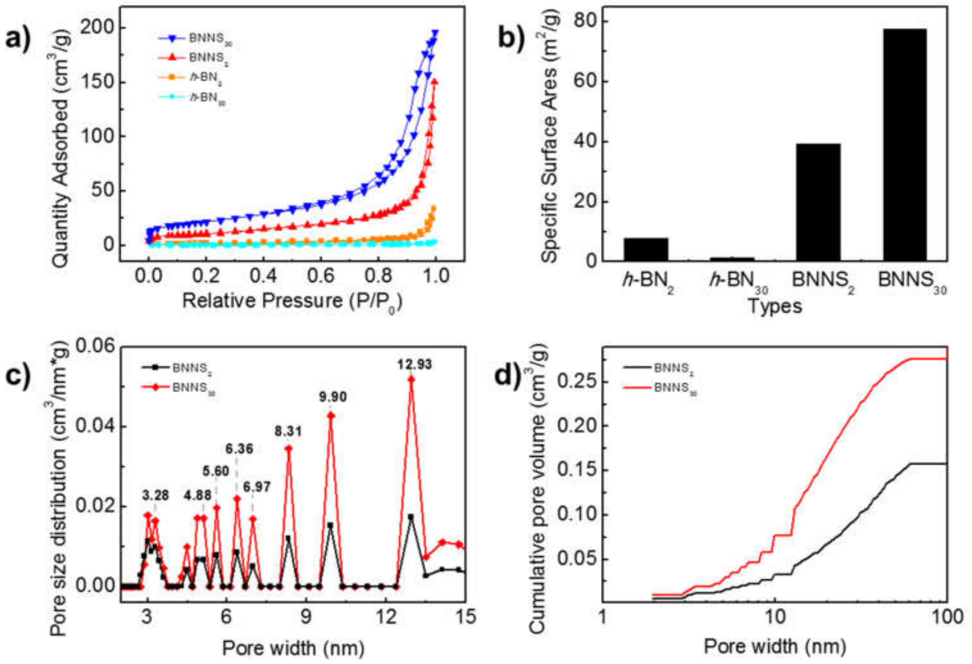

Figure S1. Nitrogen adsorption-desorption isotherms and specific surface areas of h-BN_2, h-BN_{30}, $BNNS_2$ and $BNNS_{30}$. (a) the isotherm plot. (b) the specific surface areas of h-BN raw materials and BNNSs, which are 7.55 m^2/g, 1.33 m^2/g, 39.12 m^2/g and 77.46 m^2/g for h-BN_2, h-BN_{30}, $BNNS_2$ and $BNNS_{30}$ respectively. (c) Pore size distribution(PSD) and (d) cumulative pore volume(CPV) of $BNNS_2$

and $BNNS_{30}$ obtained from nitrogen adsorption isotherms by using nonlocal density functional theory (NLDFT) with carbon slit pore model.

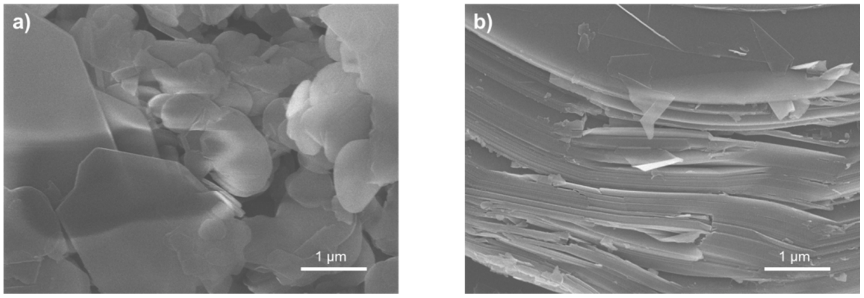

Figure S2. SEM images of (a) *h*-BN_2 and (b) *h*-BN_{30}.

While *h*-BN_2 and *h*-BN_{30} has only 7.75 and 1.33 m^2/g, after exfoliation, apparent increase in specific surface area were found for $BNNS_2$ and $BNNS_{30}$ as 39.12 and 77.46 m^2/g respectively (Figure S1a,b). It supports the successful exfoliation of *h*-BN as thinner layers provide higher specific surface area. The lower specific surface area of h-BN_{30} than *h*-BN_2 can be rationalized from the SEM observation that thickness of *h*-BN_{30} is larger than that of *h*-BN_2. According to Guo et al.'s model [1] of slot pores created during irregular graphene plate stacking in bulk graphene sample. A stack thickness X can be determined from the actual PSD stack periodicity (Fig.S1c). All of the porosity (meso- and microscale) originates from different forms of irregular stacking of the multilayer flakes as 2X, 3X, 6X. In their work, plate thickness (X) is determined to be 1.55 nm corresponding to approximately 4 graphene layers per stack, which is in agreement with the layer number range (3–5) provided by the manufacturer. In our results (Fig.S1c), it seems that $BNNS_2$ and $BNNS_{30}$ exhibit very similar peaks. X of 3.28nm is determined, which corresponds to 9–10 BN layers in a stack. This is in agreement with the observation from TEM. In turn, X of 3.28 nm provides the following pore space periodicity as 6.56nm (2X), 9.84 nm (3X), 13.12 nm (4X). The similar pore space periodicity implies both BNNS possess similar stack thickness. The much higher cumulative pore volume (Fig.S1d) indicates the higher specific surface area of $BNNS_{30}$ than $BNNS_2$.

The skeletal density is measured to be 1.70 g/cm^3 and 1.46g/cm^3 for $BNNS_2$ and $BNNS_{30}$, respectively. They are much lower than the density of bulk *h*-BN (2.27 g/cm^3, provided by manufacturer) and theoretical BNNS density (~2.1 g/cm^3). This may indicate nanoporosity in the exfoliated BNNS. However, as very small quantity of only 50 mg was used in evaluation, it may influence the data accuracy. Further verification is needed in future work.

Table S1. The data and results of cluster size calculation.

**Solvent type**	**Alcohol mole fraction/mol%**	***Viscosity/η**	****Diffusion coefficient/D**	**Calculated Radius of Cluster/*a***
NPA	5	1.60	0.481	0.283
	6	1.74	0.430	0.292
	7	1.84	0.370	0.320
	10	2.12	0.250	0.413
	14	2.35	0.149	0.623
	19	2.57	0.118	0.719
	20	2.60	0.118	0.710
	26	2.68	0.148	0.550
	30	2.67	0.167	0.487
	40	2.59	0.227	0.371
	49	2.47	0.296	0.298
	50	2.47	0.306	0.289
	60	2.33	0.375	0.249
	70	2.20	0.444	0.223

	80	2.10	0.533	0.195
IPA	5	1.73	0.560	0.226
	6	1.90	0.489	0.235
	7	2.09	0.438	0.238
	10	2.47	0.296	0.298
	14	2.74	0.185	0.430
	19	3.00	0.165	0.441
	20	3.04	0.165	0.435
	26	3.10	0.186	0.378
	30	3.07	0.196	0.362
	40	2.89	0.238	0.316
	49	2.69	0.300	0.270
	50	2.67	0.310	0.263
	60	2.44	0.393	0.227
	70	2.26	0.536	0.180
	80	2.13	0.955	0.107

* data were taken from ref [2]. ** data were taken from ref [3].

Reference

1. Guo, F.; Creighton, M.; Chen, Y.; Hurt, R.; Külaots, I. Porous structures in stacked, crumpled and pillared graphene-based 3D materials. *Carbon* **2014**, *66*, 476–484.
2. Pang, F.-M.; Seng, C.-E.; Teng, T.-T.; Ibrahim, M. Densities and viscosities of aqueous solutions of 1-propanol and 2-propanol at temperatures from 293.15 K to 333.15 K. *J. Mol. Liq.* **2007**, *136*, 71–78.
3. Pratt, K.; Wakeham, W. The mutual diffusion coefficient for binary mixtures of water and the isomers of propanol. *Proc. R. Soc. Lond. A Math. Phys. Sci.* **1975**, *342*, 401–419.

.

.
